# Imidazopyridazines as potent inhibitors of *Plasmodium falciparum* calcium-dependent protein kinase 1 (*Pf*CDPK1): Preparation and evaluation of pyrazole linked analogues^☆^

**DOI:** 10.1016/j.bmcl.2013.08.010

**Published:** 2013-11-01

**Authors:** Jonathan M. Large, Simon A. Osborne, Ela Smiljanic-Hurley, Keith H. Ansell, Hayley M. Jones, Debra L. Taylor, Barbara Clough, Judith L. Green, Anthony A. Holder

**Affiliations:** a Centre for Therapeutics Discovery, MRC Technology, Mill Hill, London NW7 1AD, UK; b Division of Parasitology, MRC National Institute for Medical Research, The Ridgeway, Mill Hill, London NW7 1AA, UK

**Keywords:** *Plasmodium falciparum*, Calcium-dependent protein kinase 1, Malaria, Imidazopyridazine, SAR

## Abstract

The structural diversity and SAR in a series of imidazopyridazine inhibitors of *Plasmodium falciparum* calcium dependent protein kinase 1 (*Pf*CDPK1) has been explored and extended. The opportunity to further improve key ADME parameters by means of lowering log D was identified, and this was achieved by replacement of a six-membered (hetero)aromatic linker with a pyrazole. A short SAR study has delivered key examples with useful in vitro activity and ADME profiles, good selectivity against a human kinase panel and improved levels of lipophilic ligand efficiency. These new analogues thus provide a credible additional route to further development of the series.

## Graphical abstract

**Figure:**
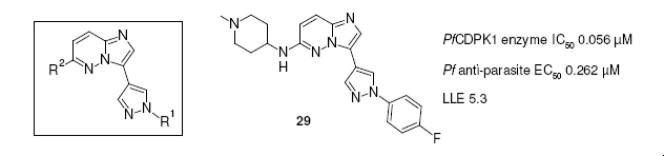

Malaria is among the most widespread and dangerous infectious diseases of the developing world, with the protozoan parasite *Plasmodium falciparum* the main causative agent. More than 3 billion people are reported to be at risk of contracting the condition, which is now thought to be responsible for approximately 1 million fatalities each year.^1^ Young children under 5 years of age and pregnant women account for a significant proportion of reported cases within large affected populations in southern Africa and south-east Asia. Widespread resistance to many small molecule malarial drugs, including the current standard-of-care Artemisinin-based combination therapy (ACT), has emerged,^2^ and this has prompted significant investment and research into urgently required new therapies and treatments.

Among at least five calcium-dependent protein kinases (CPDKs) expressed in *Plasmodium* parasites,^3^ *Plasmodium falciparum* calcium-dependent protein kinase 1 (*Pf*CDPK1)^4^ is known to be involved in key life-cycle stages of parasite motility and red blood cell invasion.^5,6^ Inhibition of the function of this enzyme is thought to represent a novel mechanism for malaria treatment.^7^ We recently reported the discovery of a series of potent and selective imidazopyridazine inhibitors of *Pf*CDPK1.^8^ These compounds, of type **1** (Fig. 1), displayed good in vitro anti-parasite activity, coupled with good selectivity against human kinases and encouraging in vivo activity. Examples bearing phenyl, pyridine (e.g. **2**) or pyrimidine linkers (A) at the 3-position of the bicyclic scaffold all generated important preliminary SAR and displayed a range of ADME properties. Our goal was to further extend the structural diversity and physicochemical profile within the series, by means of exploring alternative heteroaromatic linking motifs. We considered that pyrazole linked analogues of general structure **3** would be likely to offer useful ADME property benefits (such as lowering log P and log D),^9^ while exploring the variation in spatial positioning of an appended *N*-substituent (corresponding to the isopentylamino group in **2**). Here we disclose the preparation and evaluation of pyrazole linked analogues based on **3**, and show that these compounds display promising in vitro potency and property profiles. These efforts also contribute significant new structure activity information and provide viable options for future development of the series.

We first prepared compounds in which the aminopiperidine motif at the 6-position of the imidazopyridazine core (as in **2**) was retained, as this basic side chain had been found to provide good affinity with the target enzyme. Hence intermediates **4** or **5** were coupled with commercially available boronate esters to afford the initial targets **6**–**12** (Scheme 1, Table 1).^10^ Analogues **6** and **7** bearing secondary alkyl or cycloalkyl groups showed low potency at the target enzyme, coupled with modest levels (LE ⩽ 0.30) of ligand efficiency.^11^ We considered this value to be our minimum requirement, and sought to reach the value for **2** (LE = 0.37) where possible. A moderate 3-4 fold improvement in potency could be made by chain extension of the pyrazole alkyl substituent in **8** and **9**. However, a significant further boost in affinity was achieved by switching to a phenyl pyrazole substituent (in **10**). In contrast, methylation of the pyrazole linker in the 5-position in completely abrogated this potency gain, suggesting that an unmodified linker was optimal. These observations were confirmed by preparation of the *N*-demethylated variant **12** (LE = 0.37), although this change resulted in lower passive permeability (data not shown). Further, **10** and **12** displayed good ligand efficiency and sub-micromolar EC_50_ values in a *P. falciparum* parasite growth assay.^12^

These observations encouraged us to prepare additional substituted aryl pyrazole analogues, which could be accessed by following either of two general synthetic approaches (Scheme 2).^13^ The first involved preparation of discrete pyrazole boronate esters by means of copper-catalysed N-arylation of pyrazole **13**,^14^ followed by regioselective bromination at the 4-position of the pyrazole ring^15^ and conversion to the pinacol boronate esters of general structure **14**. Several aryl pyrazole coupling partners were prepared in this way, and could be reacted with intermediates such as **4** or **5** to provide the target compounds (see Table 2). A second more convergent route involved reaction of **4** with *N*-BOC-protected pyrazole-4-pinacol boronate; efficient in situ N-deprotection was observed under the Suzuki reaction conditions employed, affording **15** as the major product. Subsequent copper-catalysed N-arylation of this key intermediate with the appropriate aryl bromide or iodide, followed by deprotection and methylation of the amine side chain where necessary gave the desired products. Benzyl analogue **20** could be prepared by N-benzylation of pyrazole-4-boronic acid pinacol ester,^16^ followed by cross coupling of **4** as before.Introduction of fluorine atoms to aromatic rings is a well-precedented strategy in medicinal chemistry.^17^ This approach can often result in stabilisation of the aromatic ring to metabolic attack at a particular position, whilst offering control of important physicochemical parameters. Hence three isomeric monofluorophenyl analogues were prepared and evaluated; the 2- and 4-isomers (**16** and **18**, respectively—Table 2) were slightly more potent than the 3-isomer **17**, with all showing very similar levels of in vitro activity against the parasite. These observations were further probed by combining two fluorine substituents in **19**, but no additional gain in potency was obtained. Preparation of benzyl analogues such as **20** offered the opportunity to vary the spacial positioning of the aryl pyrazole substituent. One possible explanation for the resulting lower enzyme affinity^18^ is that any enthalpic gain driven by additional binding interactions had been offset by a larger entropic contribution by the additional rotatable bond. Returning to directly linked aryl pyrazoles, introduction of a 2-methyl substituent in **21** restored enzyme affinity, but despite satisfactory passive permeability (PAMPA P_app_ 110 nm s^−1^) this did not translate into a good anti-parasite EC_50_.

These compounds possessed measured log D values which were broadly similar to that of **2** (m Log D 3.4). Having attained useful levels of affinity and ligand efficiency, we wanted to make further efforts to move this key ADME parameter into more desirable property space. To this end, heterocyclic aromatic groups were appended to the pyrazole with the aim of further lowering log D, and thus improving the likelihood of more favourable microsomal stability (Table 2). Introduction of a 2-position heteroatom in pyridine analogue **22** resulted in a fivefold decrease in enzyme potency, which could be linked to repulsion between the pyridine nitrogen and the pyrazole ring’s 5-nitrogen atom, resulting in an altered conformation about the pyrazole-pyridine bond. This contrasted with a small improvement in the in vitro parasite activity for **22**, which is likely to be driven by an increase in off-target activity. These observations were supported by the 6-fluoropyridine isomer **23** and pyrimidine variants **24** and **25**, which were also revealed to be of lower enzyme potency. However, pyrazine analogue **26** showed improved affinity and in vitro anti-parasite activity, although this was accompanied by low passive permeability (PAMPA P_app_ 0.5 nm s^−1^).

We next explored variation of the 6-amino side chain as a means of adjusting the ADME properties of the series whilst maintaining potency. A number of alternative basic substituents had previously been shown to perform well in this context^8^ and key examples containing these groups were prepared and evaluated here. Regioselective nucleophilic displacement at the 6-position of diahlogenated imidazopyridazine **27**, cross coupling to give intermediates of type **28** and subsequent functionalisation of the pyrazole motif afforded **29**–**31** in good yields (Scheme 3, Table 3). Diaminocyclohexane **29** showed a good in vitro potency and metabolic stability profile, coupled with adequate passive permeability (PAMPA P_app_ 43 nm s^−1^). Cyclisation to give **30** was carried out with a view to further improving passive permeability; this was achieved (PAMPA P_app_ 104 nm s^−1^) with only a slight drop in potency, but at the expense of lower metabolic stability. The nitrogen linker atom between the basic side chain and the bicyclic scaffold was replaced with an oxygen atom in **31**. While this compound showed high passive permeability (PAMPA P_app_ 214 nm s^−1^), enzyme affinity was again slightly lower. This suggested that the presence of an N–H donor in this position was not critical for achieving good enzyme affinity, in line with earlier homology modeling studies.^8^ A further alternative involved switching to a cyclic carbon-linked side chain attached to a pyrazolopyrimidine scaffold; this closely related bicyclic template was known to show activity against this target.^19^ Hence compounds **34** and **35** were prepared by the route shown in Scheme 3,^20^ and although modest enzyme affinity was observed alongside excellent passive permeability (**34**: PAMPA P_app_ 217 nm s^−1^) and good lipophilicity (**34**: m Log D 1.4), in vitro anti-parasite activity and metabolic stability were poorer.

Returning to the original imidazopyridazine scaffold, a small number of further adjustments to the aryl pyrazole substituent were made (Table 4). Replacement of the 4-fluoro by 4-cyano in **36** resulted in a 10-fold drop in enzyme affinity, but this was partially reversed by preparing the 3-cyano analogue **37**, which also possessed good in vitro anti-parasite activity. Exchanging the amine side chain for the diaminocyclohexane gave **38**, which showed good potency against both the enzyme and the parasite^21^ coupled with excellent metabolic stability.

In the context of the series as a whole, the introduction of a pyrazole linker, in combination with an aryl or heteroaryl substituent and a basic amine side chain, has resulted in compounds with good potency and physicochemical properties. They also display key ADME properties which serve to complement analogues such as **2**, where a 6-membered aryl or heteroaryl ring linker had been used (Table 5). As expected, the pyrazole linker has contributed to lowering log D in these new analogues, and perhaps more importantly has promoted a significant increase in ligand lipophilic efficiency (LLE)^22^ as compared to compounds such as **2**. This parameter is well-known to offer a useful guide to potency and lipophilicity during the progression of chemical series from hits towards leads. In addition, we observed promising kinase selectivity profiles for key examples on screening against a human kinase panel at 1 μM concentration,^23^ as shown in Figure 2. The aryl pyrazoles described here thus represent a useful additional possibility for progressing towards alternative early lead compounds with good development characteristics.

In summary, we have shown that replacement of a six-membered aryl or heteroaryl linker motif with a pyrazole ring is a productive strategy for adding useful structural diversity to a series of imidazopyridazine *Pf*CDPK1 inhibitors. Selected examples provided a good balance of enzyme affinity and in vitro anti-parasite activity, lipophilicity and ADME properties, and this structural class holds promise for future development. Additional work on further improving the ADME property profile towards additional in vivo studies is in progress and will be reported in due course.

## Figures and Tables

**Figure 1:**
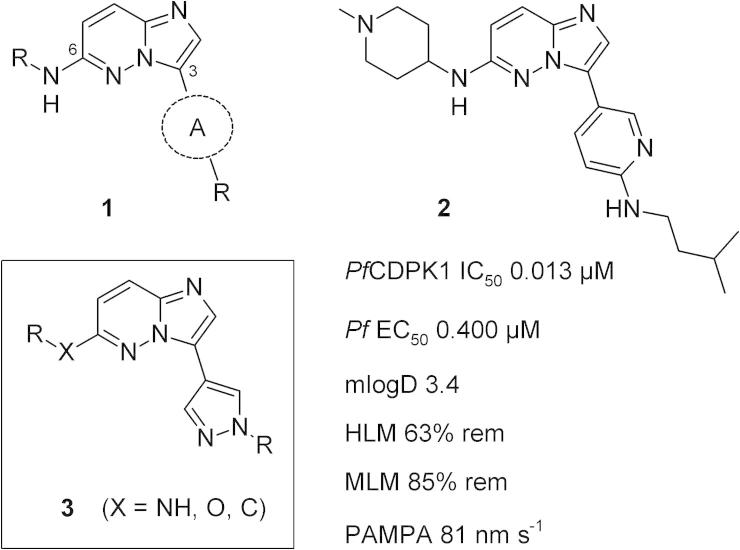
General structure of imidazopyridazines **1**, pyridine linked example **2**^8^ and pyrazole targets **3**. A = (hetero)aromatic linker. ADME data for **2**; m Log D = measured log D; HLM, MLM = % remaining after 30 min incubation with human or mouse liver microsomes; PAMPA passive permeability.

**Figure 2:**
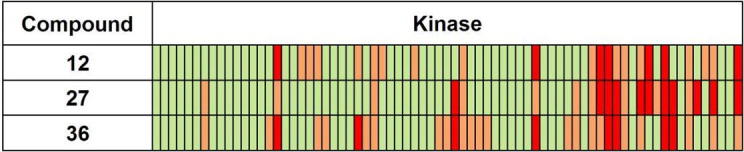
Kinase selectivity data for key aryl pyrazoles on screening against a 73-member human kinase panel at 1 μM concentration; green <50% inhibition; orange 50–80% inhibition; red >80% inhibition.^24^

**Scheme 1:**
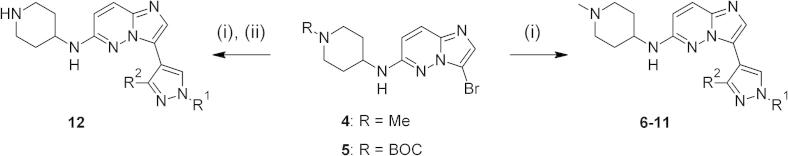
Reagents and conditions: (i) PdCl_2_(dppf), pyrazole-4-boronic acid or pincaol ester, Cs_2_CO_3_, dioxane, 90 °C; (ii) for R = BOC: 4 M HCl, dioxane, rt.

**Scheme 2:**
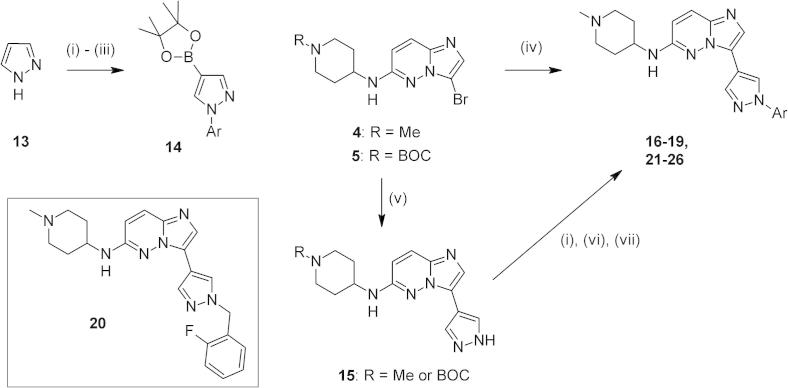
Reagents and conditions: (i) ArBr or ArI, CuI (cat), l-proline (cat), K_2_CO_3_, DMSO, 90 °C; (ii) Br_2_, AcOH, rt; (iii) PdCl_2_(dppf), B_2_pin_2_, KOAc, dioxane, 90 °C; (iv) for **4**: PdCl_2_(dppf), **14**, Cs_2_CO_3_, dioxane, 90 °C; (v) for **4** or **5**: PdCl_2_(dppf), 1-*N*-Boc-pyrazole-4-boronic acid pinacol ester, Cs_2_CO_3_, dioxane, 90 °C; (vi) 4 M HCl, dioxane, rt; (vii) HCHO, NaBH(OAc)_3_, AcOH, THF, rt.

**Scheme 3:**
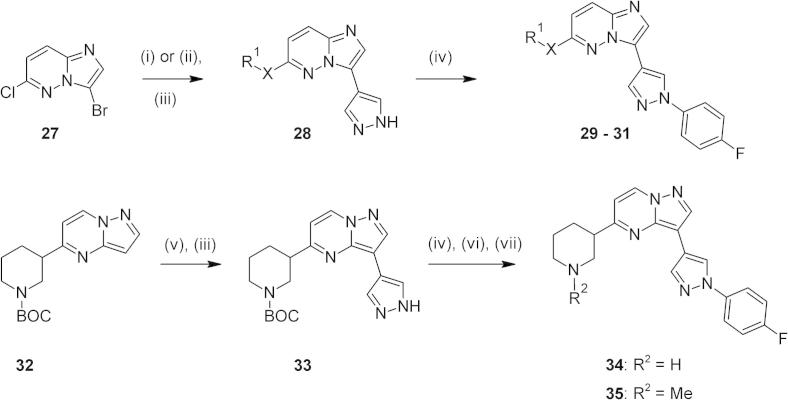
Reagents and conditions: (i) for X = NH: R^1^NH_2_, NMP, sealed tube, 180 °C; (ii) for X = O: R^1^OH, NaH, THF, 65 °C; (iii) PdCl_2_(dppf), 1-*N*-Boc-pyrazole-4-boronic acid pinacol ester, Cs_2_CO_3_, dioxane, 90 °C; (iv) 4-fluoro-1-iodobenzene, CuI (cat), l-proline (cat), K_2_CO_3_, DMF, 120 °C; (v) NBS, dibenzoyl peroxide (cat), MeCN, rt; (vi) 4 M HCl, dioxane, rt; (vii) for **35**: HCHO, NaBH(OAc)_3_, AcOH, THF, rt.

**Table 1:** Pyrazole *N*-substituent variations

Compound	R^1^	R^2^	*Pf*CDPK1 IC_50_ (μM)	*Pf* EC_50_^a^ (μM)	Compound	R^1^	R^2^	*Pf*CDPK1 IC_50_ (μM)	*Pf* EC_50_^a^ (μM)
**2**	—	—	0.013	0.400	**9**		H	0.546	*nt*
**6**		H	1.98	*nt*	**10**		H	0.071	0.999
**7**		H	1.50	*nt*	**11**		Me	3.61	*nt*
**8**		H	0.480	*nt*	**12**		H	0.043	0.985

a *nt* = not tested.

**Table 2:** Aryl and heteroaryl pyrazole SAR

**Figure:**
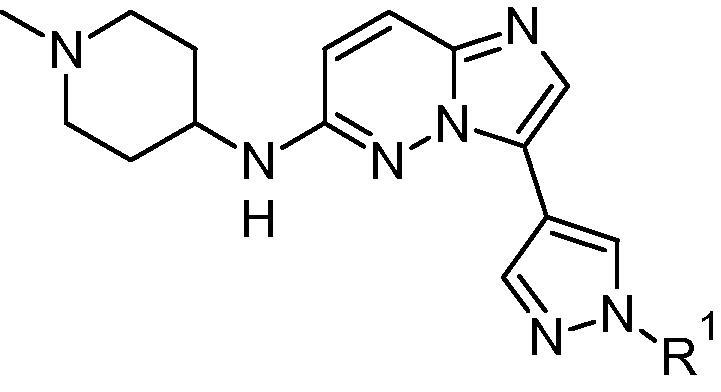

Compound	R^1^	*Pf*CDPK1 IC_50_ (μM)	*Pf* EC_50_^a^ (μM)	m Log D	Compound	R^1^	*Pf*CDPK1 IC_50_ (μM)	*Pf* EC_50_^a^ (μM)	m Log D
**16**		0.076	0.567	3.4	**21**		0.020	1.86	2.8
**17**		0.138	0.394	3.1	**22**		0.203	0.317	2.9
**18**		0.045	0.561	2.8	**23**		0.145	1.96	2.9
**19**		0.040	0.602	2.9	**24**		0.239	1.28	1.7
**20**		0.331	*nt*	3.0	**25**		0.486	*nt*	2.0
					**26**		0.031	0.366	2.2

a *nt* = not tested.

**Table 3:** Amine side chain and scaffold SAR

**Figure:**
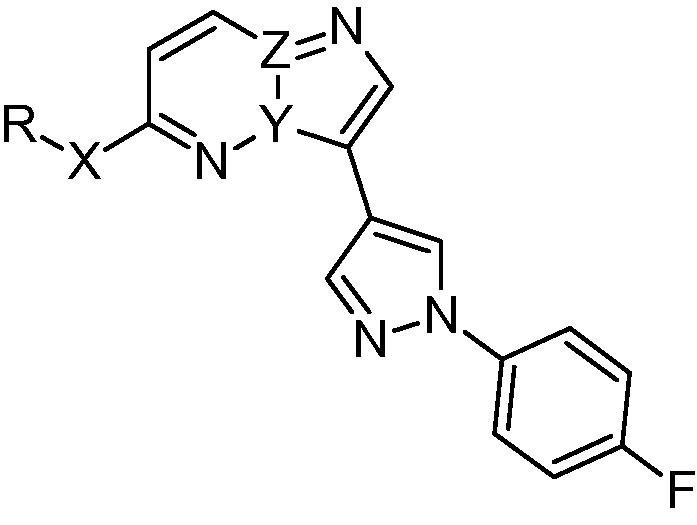

Compound	R^1^-X-	Y	Z	*Pf*CDPK1 IC_50_ (μM)	*Pf* EC_50_^b^ (μM)	m Log D	HLM % rem^c^	MLM % rem^c^
**29**		N	C	0.056	0.262	1.9	80	84
**30**		N	C	0.129	0.373	2.8	42	66
**31**		N	C	0.184	*nt*	2.6	61	38
**34**^a^		C	N	0.176	3.83	1.4	80	56
**35**^a^		C	N	0.378	3.54	2.5	55	33

a Racemate.

b *nt* = not tested.

c % Remaining after 30 min.

**Table 4:** Selected cyanophenyl pyrazoles

**Figure:**
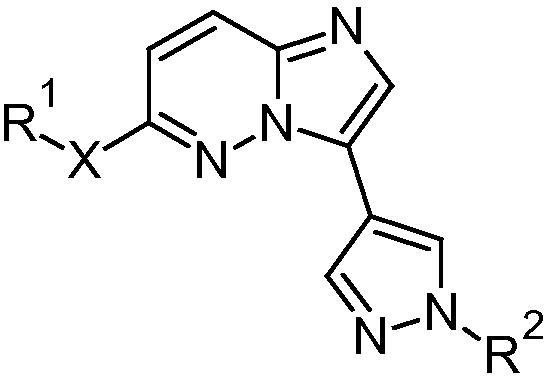

Compound	R^1^-X	R^2^	*Pf*CDPK1 IC_50_ (μM)	*Pf* EC_50_^a^ (μM)	m Log D	HLM % rem^b^	MLM % rem^b^
**29**			0.056	0.262	1.9	80	84
**36**			0.854	*nt*	1.9	79	59
**37**			0.189	0.255	2.7	70	59
**38**			0.070	0.103	1.2	93	90

a *nt* = not tested.

b % remaining after 30 min.

**Table 5:** In vitro/ADME profiles of key compounds

Compound	**12**	**26**	**29**	**38**
*Pf*CDPK1 IC_50_ (μM)	0.043	0.031	0.056	0.070
*Pf* EC_50_ (μM)	0.985	0.366	0.262	0.103
LLE^a^	4.5	5.6	5.3	6.0
MLM (% rem)^b^	74	51	84	90
HLM (% rem)^b^	81	66	80	93
m Log D	1.8	2.2	1.9	1.2
PAMPA P_app_ (nm s^−1^)^c^	23	0.5	43	*nr*

a LLE = pIC_50_ − m Log D (note that LLE for **2** = 4.5).

b % Remaining after 30 min.

c *nr* = no result.

## Supplementary data

Supplementary data This document file contains Supplementary material.

## References

[1] Murray C.J.L., Rosenfeld L.C., Lim S.S., Andrews K.G., Foreman K.J., Haring D., Fullman N., Naghavi M., Lozano R., Lopez A.D. (2012). Lancet.

[2] Petersen I., Eastman R., Lanzer M. (2011). FEBS Lett..

[3] Ward P., Equinet L., Packer J., Doerig C. (2004). BMC Genomics.

[4] Zhao Y., Kappes B., Franklin R.M. (1993). J. Biol. Chem..

[5] Green J.L., Rees-Channer R.R., Howell S.A., Martin S.R., Knuepfer E., Taylor H.M., Grainger M., Holder A.A. (2008). J. Biol. Chem..

[6] Azevedo M.F., Sanders P.R., Krejany E., Nie C.Q., Fu P., Bach L.A., Wunderlich G., Crabb B.S., Gilson P.R. (2013). Biochem. J..

[7] Lemercier G., Fernandez-Montalvan A., Shaw J.P., Kugelstadt D., Bomke J., Domostoj M., Schwarz M.K., Scheer A., Kappes B., Leroy D. (2009). Biochemistry.

[8] Chapman T.M., Osborne S.A., Bouloc N., Large J.M., Wallace C., Birchall K., Ansell K.H., Jones H.M., Taylor D., Clough B., Green J.L., Holder A.A. (2013). Bioorg. Med. Chem. Lett..

[9] Cui J.J., Tran-Dubé M., Shen H., Nambu M., Kung P.-P., Pairish M., Jia L., Meng J., Funk L., Botrous I., McTigue M., Grodsky N., Ryan K., Padrique E., Alton G., Timofeevski S., Yamazaki S., Li Q., Zou H., Christensen J., Mroczkowski B., Bender S., Kania R.S., Edwards M.P. (2011). J. Med. Chem..

[b0050] 10All compounds submitted for biological testing were of ⩾95% purity as determined by ^1^H NMR and LC–MS analysis. See Supplementary data for further details.

[11] Hopkins A.L., Groom C.R., Alex A. (2004). Drug Discovery Today.

[b0060] 12*P. falciparum* EC_50_ values were measured using an in vitro model of malaria parasite growth. Compounds were diluted into 2% DMSO and added to parasites 24 h post-invasion in a 96-well plate and incubated under static conditions. Cells were recovered 48 h later and processed for FACS analysis using hydroethidine to stain parasite DNA. Data was acquired using a Guava easyCyte Plus flow cytometer with ExpressPro software. Growth inhibition was calculated using the following formula: % growth inhibition = (1 − [parasitaemia of culture/parasitaemia of control culture]) × 100.

[b0065] 13*The two-step preparation of **17** from **4** is representative:* a solution of **4**^8^ (500 mg, 1.61 mmol) in degassed dioxane (10 mL) was treated with PdCl_2_(dppf) (0.2 equiv, 0.32 mmol, 263 mg), 1-*N*-Boc-pyrazole-4-boronic acid pinacol ester (3 equiv, 4.84 mmol, 1.42 g) and Cs_2_CO_3_ (4 equiv, 6.45 mmol, 2.10 g) and stirred at 90 °C for 18 h. Concentration in vacuo directly onto silica and flash column chromatography (2–22% 2 M NH_3_/MeOH in CH_2_Cl_2_) gave intermediate **15** (R = Me) (378 mg, 79%) as a pale brown gum; ^1^H NMR (400 MHz, DMSO-*d*_6_) 8.34 (br s, 1H), 8.13 (br s, 1H), 7.69 (s, 1H), 7.68 (d, *J* = 9.6 Hz, 1H), 6.96 (d, *J* = 6.9 Hz, 1H), 6.61 (d, *J* = 9.6 Hz, 1H), 3.70–3.60 (m, 1H), 2.83–2.77 (m, 2H), 2.21 (s, 3H), 2.12–2.04 (m, 4H), 1.55–1.45 (m, 2H); LC–MS (ES+APCI) 298 ([M+H]^+^, 100%); a solution of this intermediate (62 mg, 0.21 mmol) in anhydrous DMF (1 mL) containing 1-fluoro-3-iodobenzene (1.1 equiv, 0.23 mmol, 0.027 mL), CuI (0.3 equiv, 0.065 mmol, 12 mg) and Cs_2_CO_3_ (2.5 equiv, 0.52 mmol, 170 mg) was stirred under nitrogen at 120 °C for 18 h. The solvent was removed in vacuo and the residue passed through a metal removal cartridge (PolymerLabs, PL-Thiol, 500 mg), eluting with 3 column volumes of methanol. Concentration in vacuo and purification by preparative LC–MS gave **17** (30 mg, 36%) as an off-white solid; ^1^H NMR (400 MHz, DMSO-*d*_6_) 9.07 (s, 1H), 8.43 (s, 1H), 7.81 (s, 1H), 7.76–7.70 (m, 2H), 7.74 (d, *J* = 9.6 Hz, 1H), 7.61–7.56 (m, 1H), 7.23–7.18 (m, 1H), 7.06 (d, *J* = 6.9 Hz, 1H), 6.67 (d, *J* = 9.6 Hz, 1H), 3.79–3.70 (m, 1H), 2.83–2.79 (m, 2H), 2.19 (s, 3H), 2.12–2.04 (m, 4H), 1.57–1.47 (m, 2H); LC–MS (ES+APCI) 392 ([M+H]^+^, 100%).

[14] Zhu L., Guo P., Li G., Lan J., Xie R., You J. (2007). J. Org. Chem..

[15] Kemnitzer W., Sirisoma N., Jiang S., Kasibhatla S., Crogan-Grundy C., Tseng B., Drewe J., Cai S.X. (2010). Bioorg. Med. Chem. Lett..

[16] Yin Z., Chen Y.-L., Kondreddi R.R., Chan W.L., Wang G., Ng R.H., Lim J.Y.H., Lee W.Y., Jeyaraj D.A., Niyomrattanakit P., Wen D., Chao A., Glickman J.F., Voshol H., Mueller D., Spanka C., Dressler S., Nilar S., Vasudevan S.G., Shi P.-Y., Keller T.H. (2009). J. Med. Chem..

[17] Hagmann W.K. (2008). J. Med. Chem..

[b0090] 18A small number of further benzyl pyrazole analogues were prepared and tested, but these generally showed lower enzyme affinity (typically 100–500 nM) and in vitro anti-parasite activity (typically >1 μM).

[b0095] 19Further profiling of additional compounds based on this pyrazolopyrimidine scaffold will be disclosed in a future publication.

[20] Dwyer M.P., Paruch K., Labroli M., Alvarez C., Keertikar K.M., Poker C., Rossman R., Fischmann T.O., Duca J.S., Madison V., Parry D., Davis N., Seghezzi W., Wiswell D., Guzi T.J. (2011). Bioorg. Med. Chem. Lett..

[b0105] 21A pyrazolopyrimidine analogue of **38** bearing a C-linked piperidine side chain showed a significant loss of potency against the enzyme (*Pf*CDPK1 IC_50_ 1.1 μM).

[22] Leeson P.D., Springthorpe B. (2007). Nat. Rev. Drug Disc..

[b0115] 23Kinase selectivity profiling was carried out by the National Centre for Protein Kinase Profiling at the MRC Protein Phosphorylation Unit (University of Dundee, UK).

[b0120] 24*Kinases inhibited by **12**:* PRK2, PKD1, MSK1, MNK1, SmMLCK, CHK1, CHK2, Aurora B, NUAK1, PIM1, GCK, MINK1, MLK1, MLK3, IRAK4, SRC, LCK, YES1, EPH-A2, FGF-R1, HER4, VEG-FR. By **27**: RSK1, PRK2, CHK1, MELK, NUAK 1, PIM1, MST2, GCK, MINK1, MLK1, MLK3, IRAK4, SRC, LCK, CSK, YES1, BTK, EPH-A2, EPH-B3, HER4, VEG-FR. By 36: RSK1, ROCK2, PRK2, MNK1, MNK2, SmMLCK, PHK, CHK1, MARK3, BRSK2, MELK, NUAK1, CK1, CK2, DRYK1A, PIM1, PAK4, MST2, GCK, MINK1, MLK1, MLK3, IRAK4, SRC, YES1, BTK, EPH-A2, HER4, VEG-FR.

