# Corrigendum to “Imidazopyridazines as potent inhibitors of *Plasmodium falciparum* calcium-dependent protein kinase 1 (*Pf*CDPK1): Preparation and evaluation of pyrazole linked analogues” [Bioorg. Med. Chem. Lett. 23 (2013) 6019–6024]

**DOI:** 10.1016/j.bmcl.2013.11.012

**Published:** 2014-01-01

**Authors:** Jonathan M. Large, Simon A. Osborne, Ela Smiljanic-Hurley, Keith H. Ansell, Hayley M. Jones, Debra L. Taylor, Barbara Clough, Judith L. Green, Anthony A. Holder

**Affiliations:** a Centre for Therapeutics Discovery, MRC Technology, Mill Hill, London NW7 1AD, UK; b Division of Parasitology, MRC National Institute for Medical Research, The Ridgeway, Mill Hill, London NW7 1AA, UK

Table 4 appeared incorrectly. The corrected table appears below.

## Figures and Tables

**Table 4:** Selected cyanophenyl pyrazoles **Figure:**
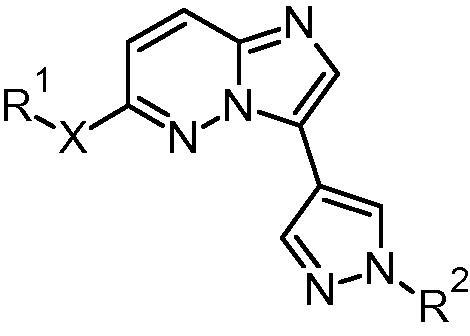

Compound	R^1^-X	R^2^	*Pf*CDPK1 IC_50_ (μM)	*Pf* EC_50_^a^ (μM)	*m* Log *D*	HLM % rem^b^	MLM % rem^b^
**29**			0.056	0.262	1.9	80	84
**36**			0.854	*nt*	1.9	79	59
**37**			0.189	0.255	2.7	70	59
**38**			0.070	0.103	1.2	93	90

a *nt* = not tested.

b % remaining after 30 min.

