# Supplementary material for: Imidazopyridazines as potent inhibitors of Plasmodium falciparum calcium-dependent protein kinase 1 (PfCDPK1): Preparation and evaluation of pyrazole linked analogues
Source: Bioorg Med Chem Lett. 2013 Nov 1;23(21):6019–24. doi: 10.1016/j.bmcl.2013.08.010 (PMC3809513; doi:10.1016/j.bmcl.2013.08.010)
Supplement: Supplementary data — This document file contains Supplementary material. [file mmc1.docx]

**Imidazopyridazines as potent inhibitors of *Plasmodium falciparum* calcium-dependent protein kinase 1 (*Pf*CDPK1): preparation and evaluation of pyrazole linked analogues**

Jonathan M. Large,^a,*^ Simon A. Osborne,^a^ Ela Smiljanic-Hurley,^a^ Keith H. Ansell,^a^ Hayley M. Jones,^a^ Debra L. Taylor,^a^ Barbara Clough,^b^ Judith L. Green,^b^ Anthony A. Holder^b^

^a^ Centre for Therapeutics Discovery, MRC Technology, Mill Hill, London, NW7 1AD, U.K.

^b^ Division of Parasitology, MRC National Institute for Medical Research, The Ridgeway, Mill Hill, London, NW7 1AA, U.K.

**Supplementary Content**

All commercial starting materials, reagents and solvents were used without further purification. Flash column chromatography was carried out using either an SP4 or Isolera-4 MPLC system (manufactured by Biotage) with pre-packed KP-silica gel cartridges (supplied by Biotage). ^1^H NMR spectra were obtained using a JEOL ECX400 spectrometer at room temperature; in all cases, NMR data were consistent with the proposed structures. Chemical shifts are given in parts-per-million referenced to residual undeuterated solvent peak, and conventional abbreviations used for multiplicity: e.g. s, singlet; d, doublet; t, triplet; q, quartet; dd, doublet of doublets; br, broad. Analytical mass spectra were recorded using a multi-mode ES+APCI mass spectrometer (6120 quadrupole LCMS manufactured by Agilent). Preparative high pressure liquid chromatography was carried out using apparatus made by Agilent. The apparatus is constructed such that the chromatography (column: 19 x 100 mm (5 µm) Prep C-18 XBridge column at a flow rate of 40 ml/min) is monitored by a multi-wavelength UV detector (G1365B manufactured by Agilent) and a multi-mode ES+APCI mass spectrometer (G-1956A, manufactured by Agilent) connected in series, and if the appropriate criteria are met the sample is collected by an automated fraction collector (G1364B manufactured by Agilent). Collection can be triggered by any combination of UV or mass spectrometry, or can be based on time. Typical conditions for the separation process are as follows: the gradient is run over a 10 minute period (gradient at start: 10% methanol and 90% water, gradient at finish: 100% methanol and 0% water). For buffering, either 0.1% trifluoroacetic acid is added to the water (low pH buffer), or 0.1% ammonium hydroxide is added to the water (high pH buffer). It may be necessary or desirable to modify the conditions for a specific compound, for example by changing the solvent composition at the start or at the end, modifying the solvents or buffers, changing the run time, changing the flow rate and/or the chromatography column. For each compound purified, the collected fraction is analysed using four methods: either methanol or acetonitrile as the organic solvent, at either low pH (0.1% TFA) or high pH (0.1% ammonium hydroxide), and if necessary re-purified until ≥ 95% purity at 254 nm was achieved. This criterion was met for all compounds submitted for biological testing. Those obtained as TFA salts were desalted by passing through an Isolute “Aminopropyl” NH_2_ cartridges (500 mg or 1 g size, eluting with three column volumes of a 2:1 CH_2_Cl_2_-MeOH solvent mixture), to give the free base before submitting for biological testing or ADME assays.

**Methods for determining ADME properties**

**LogD**

LogD measurements were carried out using the shake flask method. Compound was diluted from 10 mM DMSO stock solution into an eppendorf containing equal amounts of octanol and phosphate buffered saline (PBS) to give a final concentration of 100 μM. The tubes were shaken for 12 hours, centrifuged at 10000 rpm for 10 minutes and samples taken from the octanol and PBS layers. The samples from both layers were analysed in triplicate by LC-MS/MS (Agilent Technologies G6410 series, triple quadrupole with MM-ESI ion source) using optimised multiple reaction monitoring (MRM) scans and a standard column gradient on an Acquity UPLC BEH C8 1.7 μm column, running acetonitrile and water with 0.05% Acetic Acid as the mobile phase. The ratios of areas of the peaks were used to calculate the LogD in accordance with the equation:

LogD = Log_10_(Area-TL/Area BL)

**Kinetic Solubility**

The kinetic solubility was measured by diluting a small amount of 10 mM DMSO stock into PBS pH7.4 in a filtration plate at a target concentration of 200 μM giving a final solution composition of 98:2 PBS:DMSO. Each compound was run in triplicate on the same plate with two standard compounds, Verapamil and Ketoconazole, included per plate. The filtration plate was shaken at 500 rpm for 90 minutes and then filtered under vacuum. The filtrate was sampled and diluted with a DMSO:PBS mixture in a flat bottomed UV plate to give a solution with a composition of PBS:DMSO 80:20. A dilution series for each compound was then created in flat bottomed UV plates in PBS:DMSO 80:20 with concentrations 200 μM, 100 μM, 50 μM, 25 μM, 12.5 μM & 6.75 μM. The UV absorbance for these solutions was read across 230-400 nm at 1 nm intervals using a TECAN Safire II plate reader and a suitable UV wavelength chosen around the UV maximum of each compound. This was used to calculate the concentration in the filtrate for each compound, and hence amount remaining in solution after 90 minutes which is reported as the kinetic solubility.

**PAMPA passive permeability**

PAMPA assays were carried out using a 96 well BD Biosciences pre-coated PAMPA plate with 0.4 μm polyvinylidene fluoride filter plate, precoated with structured layers of phospholipids. The compounds were run in triplicate on the same plate with at a specified concentration between 200 μM and 50 μM solution which is known to be below the previously determined kinetic solubility, the final solution having a PBS:DMSO ratio of 98:2. The compound solution was placed in the lower ‘Donor’ well of the plate and the upper ‘Acceptor’ well of the plate was filled with PBS:DMSO 98:2 buffer containing no compound following which the plates was assembled then incubated for 5 hours at room temperature. After incubation the Donor and Acceptor wells were sampled into flat UV bottomed plates and a six fold 1:2 dilution series created for each compound starting at the appropriate top concentration for each compound. The UV absorbance for these solutions was read across 230-400 nm at 1 nm intervals using a TECAN Safire II plate reader and a suitable UV wavelength chosen around the UV maximum of each compound. This was used to calculate the concentration in the Donor and Acceptor wells for each compound. The compound permeability P_app_ (nm s^-1^) and % mass retention was then calculated for each compound in accordance with the following equations:

P_app_ (nm s^-1^) =10000000 {-ln[-C_A_(t)/C_eq_]}/[A*(1/V_D_+1/V_A_)*t]

C_eq_ = [C_D_(t)* V_D_+ C_A_(t)*V_A_]/(V_D_+V_A_)

% mass retention = 1 – [C_D_(t)*V_D_ + C_A_(t)*V_A_]/(C_0_*V_D_)]

A = Filter Area, V_D_ = Donor Well Volume, V_A_ = Acceptor Well Volume, t = incubation time (secs), C_A_(t) = Acceptor concentration at time t, C_D_(t) = Donor concentration at time t, C_0_ = Initial Donor concentration

No result was reported for compounds where % mass retention exceeded 50%.

**Microsomal turnover**

Microsomal turnover data was obtained using either human liver microsomes (HLM) or mouse liver microsomes (MLM) obtained from BD Biosciences. The compounds are pre-incubated at 37 °C for 5 minutes with the microsomes and the reaction was initiated by adding an equal volume of NADPH generating solution, also obtained from BD Biosciences. The final compound concentration in the incubation is 1 μM, and the microsomal protein concentration is 0.5 mg/ml for HLM and 0.2 mg/ml for MLM. A sample is taken at t=0 and quenched with 2x volume of ice-cold methanol containing an internal standard reference compound (carbamazepine). The reaction is agitated at 37 °C for 40 minutes (HLM) and 30 minutes (MLM) when a further sample was taken and quenched in an identical fashion. The samples were centrifuged at 10000 rpm for 10 minutes and the supernatant taken for analysis in triplicate by LC-MS/MS (Agilent Technologies G6410 series, triple quadrupole with MM-ESI ion source) using optimised multiple reaction monitoring (MRM) scans and a standard column gradient on an Acquity UPLC BEH C8 1.7μm column, running acetonitrile and water with 0.05% acetic acid as the mobile phase. The % turnover is obtained by calculating the percentage difference of the peak areas, normalised to the internal standard, at t=0 and t=40 (for human) or t=30 (for mouse). Verapamil is used as the standard compound for this assay.
